# Supplementary material for: Anti-Inflammatory Activity of Oxyresveratrol Tetraacetate, an Ester Prodrug of Oxyresveratrol, on Lipopolysaccharide-Stimulated RAW264.7 Macrophage Cells
Source: Molecules. 2022 Jun 18;27(12):3922. doi: 10.3390/molecules27123922 (PMC9228887; doi:10.3390/molecules27123922)
Supplement: Supplementary file 1 [file molecules-27-03922-s001.zip › molecules-1773179-supplementary.pdf]

molecules

MDPI

Supplementary Materials

Anti-Inflammatory Activity of Oxyresveratrol Tetraacetate, an Ester Prodrug of Oxyresveratrol, on Lipopolysaccharide-Stimulated RAW264.7 Macrophage Cells

Wuttinont Thaweesest [1,2], Visarut Buranasudja [1,3,*], Rianthong Phumsuay [1], Chawanphat Muangnoi [4], Opa Vajragupta [1,5], Boonchoo Sritularak [1,6], Paitoon Rashatasakhon [7] and Pornchai Rojsitthisak [1,8]

1 Center of Excellence in Natural Products for Ageing and Chronic Diseases, Chulalongkorn University, Bangkok 10330, Thailand; t.tinon@hotmail.com (W.T.); rianthong_p@hotmail.com (R.P.); opa.v@chula.ac.th (O.V.); boonchoo.sr@chula.ac.th (B.S.); pornchai.r@chula.ac.th (P.R.)
2 Pharmaceutical Chemistry and Natural Products Program, Faculty of Pharmaceutical Sciences, Chulalongkorn University, Bangkok 10330, Thailand
3 Department of Pharmacology and Physiology, Faculty of Pharmaceutical Sciences, Chulalongkorn University, Bangkok 10330, Thailand
4 Cell and Animal Model Unit, Institute of Nutrition, Mahidol University, Nakhon Pathom 73170, Thailand; chawanphat.mua@mahidol.ac.th
5 Molecular Probes for Imaging Research Network, Faculty of Pharmaceutical Sciences, Chulalongkorn University, Bangkok 10330, Thailand
6 Department of Pharmacognosy and Pharmaceutical Botany, Faculty of Pharmaceutical Sciences, Chulalongkorn University, Bangkok 10330, Thailand
7 Department of Chemistry, Faculty of Science, Chulalongkorn University, Bangkok 10330, Thailand; paitoon.r@chula.ac.th
8 Department of Food and Pharmaceutical Chemistry, Faculty of Pharmaceutical Sciences, Chulalongkorn University, Bangkok 10330, Thailand
* Correspondence: visarut.b@pharm.chula.ac.th

Extended discussion

Esterification is typically performed using acid, acid anhydride, or acyl chloride as the esterifying agents; pyridine, 1-methyl imidazole, or 4-dimethyl amino pyridine (DMAP) as catalysts; and pyridine, tetrahydrofuran (THF), *N*-methyl pyrrolidone, or 1,4-dioxane as solvents [24]. In this study, initial efforts for direct OXY esterification focused on the use of 4.2 eq of acid anhydride and pyridine, as summarized in Table S1 below. All reactions were performed under similar conditions, and the three tetrasubstituted OXY were found to be approximately 30% yield.

Table S1. The synthesis condition of OXY ester prodrugs.

Reaction	Reagent A	Reagent B	Catalyst	Temp.	Time	Purification Method	Final Yield
1	OXY (4.0 mmol)	Acetic Anhydride (16.8 mmol, 4.2 eq.)	Pyridine	RT	Overnight	Column chromatography (50%Acetone/ hexane)	26.1%
2	OXY (4.0 mmol)	Propionic Anhydride (16.8 mmol, 4.2 eq.)	Pyridine	RT	Overnight	Column chromatography (50%Acetone/ hexane)	32.6%
3	OXY (4.0 mmol)	Butyric Anhydride (16.8 mmol, 4.2 eq.)	Pyridine	RT	Overnight	Column chromatography (50%Acetone/ hexane)	31.2%

OXY has four OH groups; therefore, four *O*-acylated ester products can be formed, including mono-, di-, tri-, and tetra- ester OXY. Previously, resveratrol (RES), a polyphenolic compound with a similar chemical structure to OXY, was esterified with acetic, propionic, and butyric acids (0.11 eq.) by stirring at 28–30 °C under a nitrogen atmosphere for 48 h. Three types of RES ester products were formed—mono-, di-, and triesters—with a poor yield ranging from approximately 17% to 49% [24]. In addition, PEGylated RES products were synthesized using DCC and DMAP as coupling reagent and catalyst, respectively. The product consisted of mono-, di-, and tri-PEGylated RES in an approximate 1:8:1 ratio [24]. Moreover, acetylation of RES with acetyl chloride using triethylamine as a catalyst gave a crude product, which was further purified over a silica gel column to yield three different products as mono-acetoxy RES (10% yield), di-acetoxy resveratrol (4% yield), and tri-acetoxy resveratrol (22% yield) [21]. Recently, another synthesis of RES ester derivatives, including 3,4'-di-*O*-butanoyl RES, 3-*O*-butanoyl RES, 4′-*O*-butanoyl RES, 3,5,4′-tri-*O*-butanoyl RES, and 3,5-di-*O*-butanoyl RES, was observed to result in poor yields of about 19%, 36%, 4.4%, 1.5%, and 0.7%, respectively [23]. In this study, OXY-TAc synthesized using 4.2 equivalent of acid anhydride provided a yield of approximately 30%, in agreement with previous studies mentioned above. Further studies with an improved OXY-TAc yield can be expected with an increase in acid anhydride to more than 15 eq to ensure esterification of all phenolic functional groups [22,63].

Supplementary Figure

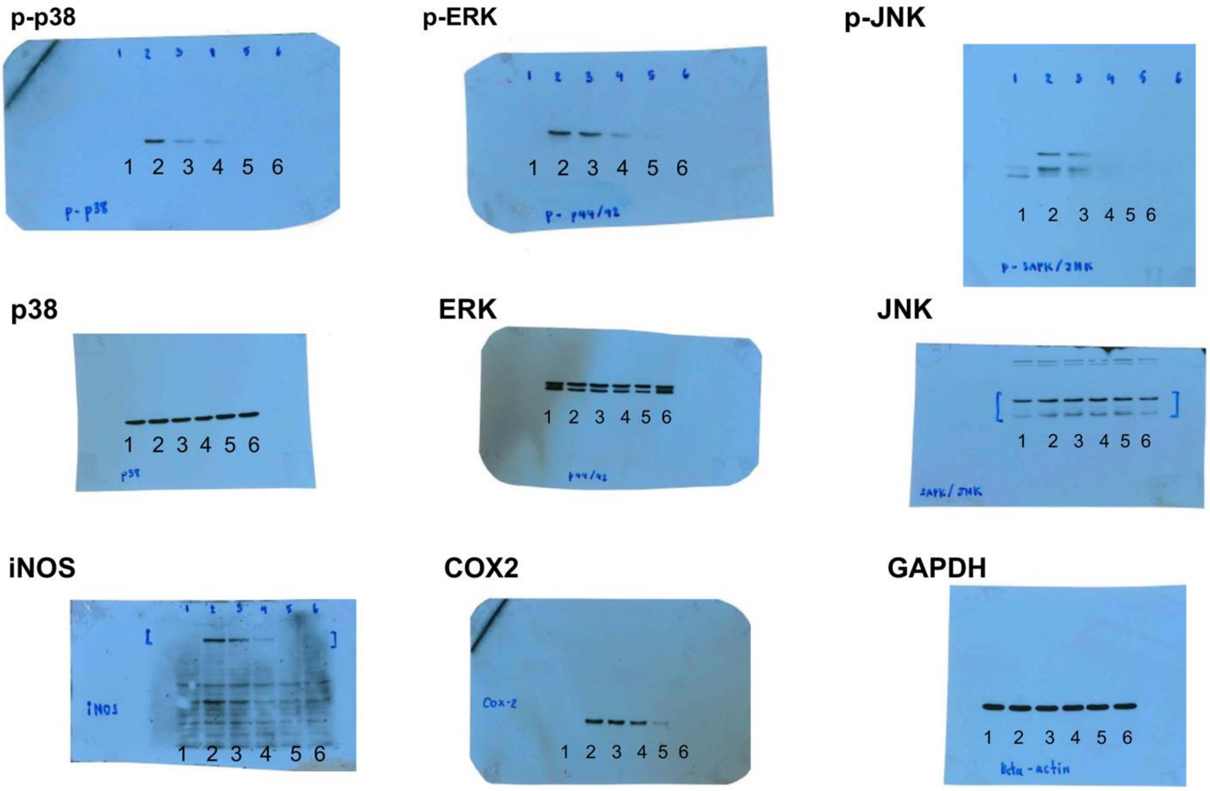

Figure S1. Full unedited western blots for p-p38, p38, p-ERK, ERK, p-JNK, JNK, iNOS, COX2, and GAPDH. Lane 1, untreated control; Lane 2, LPS; Lane 3, OXY (50 µM) + LPS; Lane 4, OXY-TAc (50 µM) + LPS; Lane 5, OXY (50 µM); Lane 6, OXY-TAc (50 µM). These western blot images represent three biological replicates and were used to prepare Figure 5.
